# Impact of early palliative care according to baseline symptom severity: Secondary analysis of a cluster‐randomized controlled trial in patients with advanced cancer

**DOI:** 10.1002/cam4.4565

**Published:** 2022-02-09

**Authors:** Rebecca Rodin, Nadia Swami, Ashley Pope, David Hui, Breffni Hannon, Lisa W. Le, Camilla Zimmermann

**Affiliations:** ^1^ Department of Medicine University of Toronto Toronto Ontario Canada; ^2^ Department of Supportive Care, Princess Margaret Cancer Centre University Health Network Toronto Ontario Canada; ^3^ Department of Palliative Care, Rehabilitation, and Integrative Medicine, and Department of General Oncology The University of Texas MD Anderson Cancer Center Houston Texas USA; ^4^ Department of Biostatistics, Princess Margaret Cancer Centre University Health Network Toronto Ontario Canada; ^5^ Department of Psychiatry University of Toronto Toronto Ontario Canada; ^6^ Princess Margaret Research Institute, Princess Margaret Cancer Centre University Health Network Toronto Ontario Canada

**Keywords:** cancer, palliative care, quality of life, randomized controlled trial, secondary analysis, symptom assessment

## Abstract

**Background:**

Early palliative care (EPC) improves the quality of life but may not be feasible for all patients with advanced cancer. Symptom screening has been suggested to triage patients for EPC, but scant evidence exists for this practice.

**Methods:**

We conducted a subgroup analysis of a cluster‐randomized controlled trial of EPC vs. standard oncology care according to patients' baseline symptom scores (high [>23] vs. low [≤23] Edmonton Symptom Assessment System Distress Score [ESAS SDS]). A linear mixed‐effects model was used to account for correlation within clusters, adjusting for the baseline outcome score and all covariates in the original trial.

**Results:**

Among the 461 participants, baseline symptom scores were high in 229 patients (127 intervention, 102 control) and low in 232 (101 intervention and 131 control). Among those with high baseline symptoms, there was improved quality of life in the EPC arm compared to controls at 4 months (adjusted difference in primary outcome of FACIT‐Sp change score [95% CI], 8.7 [2.8 to 14.5], *p* = 0.01; adjusted difference in QUAL‐E, 4.2 [0.9–7.5], *p* = 0.02); there was also improved satisfaction with care (6.9 [3.8–9.9], *p* = 0.001) and clinician‐patient interactions (−1.7 [−3.4 to −0.1], *p* = 0.04), but no significant difference in ESAS SDS (−5.6 [−12.7 to 1.4], *p* = 0.11). In the low baseline symptom group, there were no significant differences between arms for any outcomes.

**Conclusion:**

EPC improved quality of life, satisfaction with care, and clinician‐patient interactions only in those with high baseline symptoms. Symptom severity may be an appropriate criterion to trigger early referrals to palliative care.

## 1 INTRODUCTION

Patients with advanced cancer suffer from multiple physical and psychosocial symptoms.^1^, ^2^ Randomized controlled trials have demonstrated that integration of specialized palliative care within oncology early in the course of disease improves quality of life and symptom burden.^3^, ^4^, ^5^, ^6^ However, universal implementation of early specialized palliative care is not feasible, given the shortage of specialized palliative care clinicians.^7^, ^8^ A more realistic model may be one in which basic palliative care needs are provided by oncologists, while complex problems are addressed by palliative care specialists.^9^

Routine symptom screening is increasingly being implemented at cancer centers and may provide a means of identifying patients who would benefit from specialized palliative care.^10^, ^11^, ^12^ Risk‐stratifying patients according to symptoms may lead to a more equitable distribution of specialized palliative care resources to those with the greatest need and with the greatest potential for benefit. Further, automatic triggering of palliative care referrals based on such criteria could help to diminish stigma and increase the acceptance of palliative care by both patients and providers.^13^, ^14^

We previously reported on the results of a cluster‐randomized controlled trial that examined the impact of early palliative care provided in a specialized palliative care clinic on multiple patient‐reported outcomes.^3^ Compared to those receiving standard oncology care, patients assigned to the early palliative care group had improved quality of life, symptom control, and satisfaction with care 4 months after randomization. There was no difference between the intervention and standard care arms in difficulties with clinician‐patient interactions.

The aim of the current analysis was to examine whether outcomes of this cluster‐randomized trial differed between subgroups according to symptom severity at baseline. We hypothesized that early palliative care would be associated with greater improvements in quality of life, symptom control, satisfaction with care, and clinician–patient interactions in those with high symptom burden at baseline, compared to those with low symptom burden at baseline.

## 2 METHODS

### 2.1 Study design and participants

We conducted a cluster‐randomized controlled trial comparing specialized early palliative care to standard oncology care in patients with advanced cancer. The trial was conducted between December 1, 2006, and February 28, 2011, at the Princess Margaret Cancer Centre, a comprehensive cancer center in Toronto, Canada. Patients provided written informed consent to participate and the study was approved by the Research Ethics Board of the University Health Network.

Twenty‐four medical oncology clinics from breast, gastrointestinal, genitourinary, lung, or gynecological tumor sites were cluster‐randomized according to tumor site and clinic size in a 1:1 ratio using a computer‐generated sequence by a statistical team at Western University. Patients were approached for consent to participate in one of the two study arms (unaware of the other group), depending on the randomization of the clinic. Blinding of medical oncologists or study investigators was not possible.

Patients eligible to participate in the trial were at least 18 years old, had stage IV cancer (or breast or prostate cancer refractory to hormonal therapy); an estimated survival of 6–24 months, as determined by their primary oncologist; and an Eastern Cooperative Oncology Group (ECOG) performance score of 0, 1, or 2. Participants were recruited from 24 oncology clinics. Those with insufficient English literacy or cognitive impairment (Short Orientation‐Memory‐Concentration Test score <20 or >10 errors) were excluded from the study. The early palliative care intervention has been described in detail elsewhere.^3^, ^15^ It consisted of the following main components: (1) a comprehensive, 60–90 min, multidisciplinary, in‐person assessment within 1 month of recruitment, focusing on symptoms, psychological distress, social support, and home services; (2) routine telephone contact from a palliative care nurse 1 week after the first consultation, and thereafter as needed; (3) monthly outpatient palliative care follow‐up (20–50 min); and (4) a 24‐h on‐call service for telephone management of urgent issues. Additional interventions were provided depending on the status of the patient, including arrangement of home nursing or palliative physician care or transfer to the Princess Margaret Cancer Centre palliative care inpatient unit.

Data on demographic factors, treatment status, Charlson Comorbidity Index (CCI), and ECOG performance status were collected at baseline. Participants completed measures at baseline and 4 months after randomization to assess the quality of life, symptom severity, satisfaction with care, and clinician‐patient interactions.

Quality of life was assessed using two measures that evaluated different domains. The Functional Assessment of Chronic Illness Therapy—Spiritual Well‐Being (FACIT‐Sp) includes physical, social and family, emotional, functional, and spiritual domains of quality of life, with scores ranging from 0 to 156 (higher scores representing better quality of life).^16^, ^17^ The Quality of Life at the End of Life (QUAL‐E) evaluates domains of life completion, symptoms, relationship with healthcare provider, and preparation for the end of life. The total scores range from 21 to 105, with higher scores indicating better quality of life.^18^

The Edmonton Symptom Assessment System (ESAS) is a measure of symptom burden that includes a Likert rating of nine symptoms (pain, fatigue, drowsiness, nausea, anxiety, depression, appetite, dyspnea, and wellbeing) on a scale from 0 (best) to 10 (worst).^19^ The nine individual symptom scores are summed to yield the ESAS Symptom Distress Score (ESAS SDS), ranging from 0 to 90, with higher scores indicating worse symptom burden.^20^ The FAMCARE‐P16 patient satisfaction with care measure assesses satisfaction with care, including information‐giving, availability of care, psychological care, and physical care in patients with advanced cancer, with scores ranging from 16 to 80; higher scores indicate greater satisfaction with care.^21^, ^22^ The Cancer Rehabilitation Evaluation System Medical Interaction Subscale (CARES‐MIS) evaluates specific problems of patients in their interactions with nurses and doctors, including those related to information seeking and communication with the medical team (range 0–44); higher scores indicate greater difficulties with interactions.^23^

The primary outcome for the present analysis was change in patient‐reported FACIT‐Sp scores at 4 months. Secondary outcomes were change in patient‐reported QUAL‐E, ESAS SDS, FAMCARE‐P16, and CARES‐MIS scores at 4 months compared to baseline. We hypothesized that patients with greater symptom burden at baseline would have greater improvement in quality of life and other outcomes after receiving the early palliative care intervention compared to those with low baseline symptom burden.

### 2.2 Data analysis

Sample size justification for the original trial was reported previously.^3^ We grouped patients according to their baseline ESAS SDS scores, relative to the median: the high baseline symptom group was defined by those with baseline ESAS SDS scores above the median and the low baseline symptom group by those with baseline ESAS SDS scores at or below the median. A median split was used to determine these subgroups in order to maximize power for the subgroup analyses. The primary outcome of FACIT‐Sp, as well as the secondary outcomes of QUALE‐E, ESAS SDS, FAMCARE‐P16, and CARES‐MIS, was assessed within the high and low symptom subgroups based on the change in score at 4 months from baseline.

A linear mixed‐effects model was used for all analyses to account for the correlation within clusters, adjusting for the corresponding baseline quality of life score and all baseline covariates in the original randomized trial, including age, tumor site, baseline ECOG score, and receiving chemotherapy.^3^ Interaction between study arm and baseline symptom group was examined for each outcome by using the appropriate product term in the mixed‐effect model. In addition, the mean observed change from baseline was compared between intervention and standard care groups within each baseline symptom subgroup; adjusted differences in change scores between study arms and their associated 95% confidence intervals (CI) were reported for each baseline symptom group. Similar to the analysis for the original trial, all analyses were by intention to treat, and sensitivity analyses were performed using Markov Chain Monte Carlo (MCMC) multiple imputation for missing values.^3^ All analyses were performed using SAS statistical software (version 9.4) and two‐sided *p*‐values below 0.05 were considered significant.

## 3 RESULTS

**FIGURE 1:**
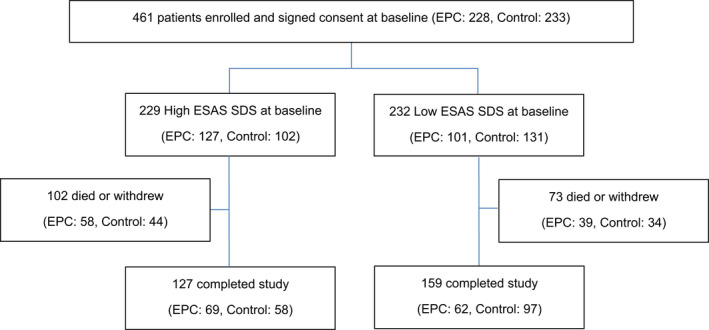
Study design

The median age of all trial participants (*n* = 461; 228 intervention, 233 control) was 61 years and the median baseline ESAS SDS score was 23 (range 2–78, interquartile range 13–36). Of the 461 participants, 229 (127 intervention and 102 control) had baseline ESAS SDS scores above the median (high baseline symptom group) and 232 (101 intervention and 131 control) had baseline ESAS SDS scores at or below the median (low baseline symptom group) (Figure 1).

The mean (SD) baseline ESAS SDS was 38.5 (11.1) in the high baseline symptom group and 12.7 (6.4) in the low baseline symptom group. The baseline characteristics of patients in the intervention and control arms, for those with high and low baseline symptoms, are shown in Table 1. For those with high baseline symptoms, there was no significant difference in baseline demographic characteristics or outcome measures between the intervention and control arms. For those with low baseline symptoms, patients in the intervention arm were more likely than those in the control arm to have worse baseline ESAS SDS, FAMCARE‐P16, and CARES‐MIS scores (Table 1).

**TABLE 1:** Baseline characteristics by symptom subgroup and study arm

	Low baseline symptoms			High baseline symptoms		
Baseline characteristic no. (%)	Intervention *n* = 101	Control *n* = 131	*p*	Intervention *n* = 127	Control *n* = 102	*p*
Age, mean (± SD)	61.6 (11.8)	61.4 (12.1)	0.84	61.0 (12.2)	58.6 (10.2)	0.11
Female	50 (49.5)	62 (47.3)	0.74	42 (33.1)	46 (45.1)	0.06
Married	72 (71.3)	91 (69.5)	0.76	84 (66.1)	76 (74.5)	0.17
Living alone	18 (17.8)	27 (20.6)	0.59	25 (19.7)	15 (14.7)	0.32
Employment status			0.21			0.20
Retired	49 (48.5)	64 (48.9)		55 (43.3)	37 (36.3)	
Employed	22 (21.8)	38 (29.0)		23 (18.1)	21 (20.6)	
Unemployed	9 (8.9)	14 (10.7)		20 (15.7)	10 (9.8)	
Disability	21 (20.8)	15 (11.5)		29 (22.8)	34 (33.3)	
Education			0.52			0.12
Below high school	7 (7.0)	7 (5.3)		11 (8.7)	17 (16.8)	
High school	22 (22.0)	37 (28.2)		34 (27.0)	20 (19.8)	
College/university/other	71 (71.0)	87 (66.4)		81 (64.3)	64 (63.4)	
Missing	1	0		1	1	
Tumor Site			0.06			0.12
Breast	13 (12.9)	20 (15.3)		28 (22.0)	11 (10.8)	
Gastrointestinal	46 (45.5)	39 (29.8)		28 (22.0)	26 (25.5)	
Genitourinary	13 (12.9)	33 (25.2)		14 (11.0)	18 (17.6)	
Gynecological	12 (11.9)	20 (15.3)		19 (15.0)	20 (19.6)	
Lung	17 (16.8)	19 (14.5)		38 (29.9)	27 (26.5)	
Active systemic cancer treatment	72 (71.3)	105 (80.2)	0.12	102 (80.3)	76 (74.5)	0.29
Radiation treatment within 14 days	5 (5.0)	4 (3.1)	0.46	11 (8.7)	9 (8.8)	0.97
CCI score >0^a^	33 (32.7)	47 (35.9)	0.61	42 (33.1)	24 (23.5)	0.11
ECOG performance status^b^			0.88			0.71
0	39 (38.6)	54 (41.2)		22 (17.3)	22 (21.6)	
1	58 (57.4)	73 (55.7)		91 (71.7)	70 (68.6)	
2	4 (4.0)	4 (3.1)		14 (11.0)	10 (9.8)	
Baseline mean FACIT‐Sp scores (± SD)^c^	112.5 ± 16.7	113.8 ± 14.2	0.78	91.4 ± 18.0	92.6 ± 17.3	0.89
Baseline mean QUAL‐E scores (± SD)^d^	77.4 ± 10.8	79.0 ± 10.5	0.21	68.8 ± 10.0	68.6 ± 10.0	0.99
Baseline mean ESAS SDS scores (± SD)^e^	13.9 ± 6.3	11.9 ± 6.3	0.01	38.7 ± 11.2	38.2 ± 11.1	0.71
Baseline mean FAMCARE‐P16 scores (± SD)^f^	66.0 ± 9.9	70.0 ± 9.0	0.002	63.3 ± 9.4	65.0 ± 9.9	0.10
Baseline mean CARES‐MIS scores (± SD)^g^	4.0 ± 5.3	2.6 ± 3.7	0.04	5.2 ± 5.8	5.7 ± 6.7	0.8

Abbreviations: CARES‐MIS, cancer rehabilitation evaluation system medical interaction subscale; CCI, charlson comorbidity index; ECOG, eastern cooperative oncology group; ESAS SDS, edmonton symptom assessment system symptom distress score; FACIT‐Sp, functional assessment of chronic illness therapy—spiritual well‐being scale; FAMCARE‐P16, FAMCARE patient satisfaction with care measure; QUAL‐E, quality of life at the end of life scale.

^a^ The CCI is a measure of comorbidity for patients with cancer; higher scores indicate more severe comorbid conditions.

^b^ An ECOG performance status score of 0 = fully active at pre‐disease performance; 1 = ambulatory but restricted in physically strenuous activity; 2 = not fully ambulatory but lying or sitting <50% of the day.

^c^ The FACIT‐Sp scale ranges from 0 to 156; higher numbers indicate better quality of life.

^d^ The QUAL‐E scale ranges from 21 to 105; higher numbers indicate better quality of life.

^e^ The FAMCARE‐P16 scale ranges from 16 to 80; higher numbers indicate better patient satisfaction with care.

^f^ The ESAS SDS scale ranges from 0 to 90; higher numbers indicate worse symptom burden.

^g^ The CARES‐MIS subscale ranges from 0 to 44; higher numbers indicate greater problems with medical interactions.

**FIGURE 2:**
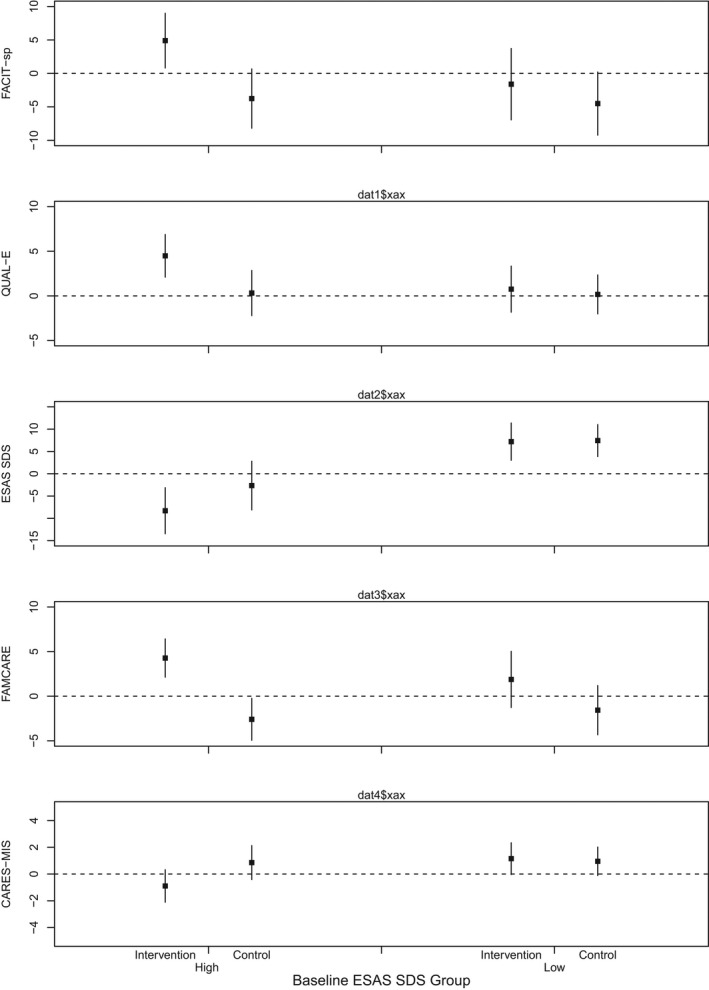
Estimated adjusted mean change in FACIT‐Sp, QUAL‐E, ESAS SDS, FAMCARE‐P16, and CARES‐MIS total scores in high and low baseline symptom subgroups at 4 months

As shown in Table 2 and Figure 2, among participants who received early palliative care, those in the high baseline symptom group had a significant improvement in FACIT‐Sp scores at 4 months compared to controls (adjusted mean difference in change scores +8.7; 95% CI 2.8–14.5, *p* = 0.01). There was no improvement in the low baseline symptom subgroup (+2.9; 95% CI −3.7 to 9.5, *p* = 0.36); the interaction term was not significant (*p* for interaction, 0.16). Similar results were demonstrated for the QUAL‐E score, which was significantly improved in those in the high baseline symptom group who received early palliative care compared to those who received standard care (adjusted mean difference [AMD] in change scores +4.2; 95% CI 0.9–7.5, *p* = 0.02). There was no significant improvement in QUAL‐E scores among those in the low baseline symptom group who received the intervention compared to those who received standard care (+0.6; 95% CI −2.6 to 3.7, *p* = 0.59). For ESAS SDS scores, there was no significant difference between early palliative care and standard care arms in those with high baseline symptoms (AMD −5.6, 95% CI −12.7 to 1.4, *p* = 0.11), nor in those with low baseline symptoms (−0.2, 95% CI −5.3 to 4.9, *p* = 0.92).

**TABLE 2:** Estimated adjusted mean changes in quality‐of‐life scores (FACIT‐Sp, QUAL‐E) and symptom burden (ESAS SDS) in high and low baseline symptom subgroups at 4 months

	Intervention		Control			
	*n*	Observed change from baseline Mean (SD)	*n*	Observed change from baseline Mean (SD)	Adjusted difference in change scores between study arms (95% CI)	*p*
FACIT‐Sp total score						0.16^a^
High baseline symptoms	66	5.1 (13.3)	56	−3.0 (15.2)	8.7 (2.8 to 14.5)^b^	0.01
Low baseline symptoms	56	−0.7 (17.3)	93	−4.5 (13.6)	2.9 (−3.7 to 9.5)^b^	0.36
QUAL‐E total score						0.09^a^
High baseline symptoms	65	4.5 (7.7)	57	0.3 (6.8)	4.2 (0.9 to 7.5)^c^	0.02
Low baseline symptoms	56	1.3 (8.8)	91	−1.0 (8.1)	0.6 (−2.6 to 3.7)^c^	0.59
ESAS SDS score						0.10^a^
High baseline symptoms	69	−7.9 (14.2)	58	−3.4 (14.4)	−5.6 (−12.7 to 1.4)^d^	0.11
Low baseline symptoms	62	6.0 (14.7)	97	7.2 (12.0)	−0.2 (−5.3 to 4.9)^d^	0.92

Abbreviations: ESAS SDS, edmonton symptom assessment system symptom distress score; FACIT‐Sp, functional assessment of chronic illness therapy—spiritual well‐being scale; QUAL‐E, quality of life at the end of life scale.

Sensitivity analyses using MCMC multiple imputation: for FACIT‐Sp, the adjusted difference in change scores was 6.5 (95% CI, 0.6–12.3; *p* = 0.03) for high baseline symptom group vs. 3.5 (−1.39 to 8.36; *p* = 0.16) for low baseline symptom group, *p* for interaction = 0.38; for QUAL‐E, 3.6 (0.5–6.7; *p* = 0.02) for high baseline symptom group vs. 1.9 (−0.5 to 4.3; *p* = 0.12) for low baseline symptom group, *p* for interaction = 0.37; and for ESAS SDS, −4.2 (−9.7 to 1.4; *p* = 0.14) for high baseline symptom group vs. ‐0.9 (−5.3 to 3.5; *p* = 0.70) for low baseline symptom group, *p* for interaction = 0.33.

^a^ *p*‐value for interaction between high/low baseline symptom subgroups and study arm.

^b^ Adjusted for age, tumor site, baseline ECOG score, receiving systemic cancer treatment, and baseline FACIT‐Sp total score.

^c^ Adjusted for age, tumor site, baseline ECOG score, receiving systemic cancer treatment, and baseline QUAL‐E total score.

^d^ Adjusted for age, tumor site, baseline ECOG score, receiving systemic cancer treatment, and baseline ESAS SDS score.

Results for the FAMCARE‐P16 and CARES‐MIS outcomes are shown in Table 3 and Figure 2. Among those assigned to early palliative care, those in the high baseline symptom group had a significant improvement in FAMCARE‐P16 at 4 months compared to controls (AMD +6.9; 95% CI 3.8–9.9, *p* = 0.001), whereas there was no significant improvement in the low baseline symptom group (+3.4; 95% CI −0.5 to 7.4, *p* = 0.08). Similarly, the high baseline symptom group reported improved clinician‐patient interactions (AMD for CARES‐MIS −1.7; 95% CI −3.4 to −0.1, *p* = 0.04), whereas there was no improvement in the low baseline symptom group (+0.2; 95% CI −1.3 to 1.7, *p* = 0.77). The *p* for interaction was 0.06 for CARES‐MIS and 0.13 for FAMCARE‐P16.

**TABLE 3:** Estimated adjusted mean change in FAMCARE‐P16 and CARES‐MIS total scores in high and low baseline symptom subgroups at 4 months

	Intervention		Control			
	*n*	Observed change from baseline	*n*	Observed change from baseline	Adjusted difference in change scores between study arms (95% CI)	*p*
		Mean (SD)		Mean (SD)		
FAMCARE‐P16 total score						0.13^a^ ^,^ ^c^
High baseline symptoms	64	4.3 (8.2)	57	−3.3 (8.3)	6.9 (3.8 to 9.9)^b^	0.001
Low baseline symptoms	57	3.1 (9.0)	96	−1.9 (8.4)	3.4 (−0.5 to 7.4)^b^	0.08
CARES‐MIS total score						0.06^a^
High baseline symptoms	64	−0.8 (4.7)	58	1.0 (4.3)	−1.7 (−3.4 to −0.1)^d^	0.04
Low baseline symptoms	59	0.1 (4.0)	96	0.4 (3.1)	0.2 (−1.3 to 1.7)^e^	0.77

Abbreviations: CARES‐MIS, cancer rehabilitation evaluation system medical interaction subscale; FAMCARE patient satisfaction with care measure.

Sensitivity analyses using MCMC multiple imputation: for FAMCARE‐P16, the adjusted difference in change scores was 5.9 (2.5–9.2; *p* < 0.001) for high baseline symptom group vs. 4.3 (1.5–7.2; *p* = 0.004) for low baseline symptom group, *p* for interaction = 0.34; for CARES‐MIS, −1.0 (−2.6 to 0.7; *p* = 0.25) for high baseline symptom group vs. ‐0.1 (−1.3 to 1.1; *p* = 0.85) for low baseline symptom group, *p* for interaction = 0.27.

^a^ *p*‐value for interaction between high/low baseline symptom subgroups and study arm.

^b^ Adjusted for age, tumor site, baseline ECOG score, receiving systemic cancer treatment, and baseline FAMCARE‐P16 total score.

^c^ Adjusted for baseline FAMCARE‐P16 score to achieve model convergence.

^d^ Adjusted for age, baseline ECOG score, receiving systemic cancer treatment, and baseline CARES‐MIS total score to achieve model convergence.

^e^ Adjusted for age, tumor site, receiving systemic cancer treatment, ECOG scores, and baseline CARES‐MIS total score.

In sensitivity analyses using MCMC imputation, there were significant differences between intervention and control groups for those with high, but not low, baseline symptoms in the primary outcome of FACIT‐Sp as well as QUAL‐E, and nonsignificant results for ESAS SDS regardless of baseline symptom severity. For satisfaction with care and clinician‐patient interactions, those who received early palliative care had improved FAMCARE‐P16 scores within both the high and low symptom groups, whereas CARES‐MIS scores did not improve significantly for those in either symptom group.

## 4 DISCUSSION

The present subgroup analysis extends the findings of a cluster‐randomized controlled trial in patients with advanced cancer^3^ by demonstrating that early palliative improved quality of life, satisfaction with care, and clinician‐patient interactions at 4 months only in those with high baseline symptoms. For symptom control, which showed a marginal benefit from early palliative care in the original trial, there was no difference between the intervention and control arms in either the high or low baseline symptom group. In sensitivity analyses, the results were consistent for quality of life and symptom control, whereas for satisfaction with care those who received early palliative care benefitted regardless of symptom group; for clinician‐patient interactions, no benefit was demonstrated in either symptom group.

The differential improvement from early palliative care in two quality of life measures among those with high, but not low, baseline symptoms is particularly striking given the lack of similar findings for symptom control. There are several possible reasons for these findings. Although the physical domain is important for quality of life, high baseline symptom burden may be a marker of greater palliative care needs across a range of other quality‐of‐life domains. Specialized palliative care not only addresses symptoms,^20^ but also strengthens coping, provides holistic support for patients and their caregivers; provides guidance in decision‐making; and assists patients in preparing for the future.^24^, ^25^, ^26^, ^27^ These interventions may lead to improvements in non‐physical domains of quality of life, such as social and spiritual wellbeing, life completion, end‐of‐life preparation, and relationships with healthcare providers. The need for interventions to address these domains tends to increase with disease severity,^27^ as does symptom burden.^2^, ^28^ Thus symptoms may provide a marker for patients who require more care in all quality‐of‐life domains.

There are also several potential explanations for the lack of differential improvement in symptom burden. Although symptom control is a core domain of early palliative care,^15^, ^24^, ^25^ symptoms were mild in the total study sample at baseline. This ceiling effect, combined with reduced sample size and power for the subgroup analysis, may have made detection of symptom improvement difficult. Of note, in the high symptom group, there was a nonsignificant trend toward greater improvement of symptoms in the early palliative care arm than in controls, whereas, in the low symptom group, symptom control tended to worsen in both trial arms. Regression to the mean may have played a role in the trends for both high and low symptom groups, though the tendency for difference between arms in the high symptom group is noteworthy.^29^ Prior studies have shown that specialized palliative care improves symptom control,^6^, ^10^ which is addressed in up to 75% of early palliative care visits.^24^

The more robust findings for patient satisfaction with care compared to clinician‐patient interactions in the current study were also demonstrated in the analysis for the original trial,^3^ despite the fact that items for the FAMCARE‐P16 and CARES‐MIS measures are similar. Indeed, for satisfaction with care, there was a tendency for improvement associated with early palliative care in both the high and low symptom groups, although the high symptom group improved to a greater extent. For clinician‐patient interactions, there was a much smaller effect only for those in the high symptom group, which was nonsignificant in the sensitivity analysis. These differences in results between similar measures may relate to the phrasing of items: FAMCARE‐P16 items emphasize the healthcare provider and are phrased positively (e.g. “Doctor's attention to your description of symptoms”; “The availability of doctors to answer your questions”), whereas CARES‐MIS items are phrased negatively and emphasize the patient (e.g. “I have difficulty telling my doctor about new symptoms”; “I have difficulty asking doctors questions”). Perhaps due to this wording, CARES‐MIS results were highly skewed, without much room for improvement. The FAMCARE‐P16 also includes several items related to symptom control, including satisfaction with pain relief, information provided on how to treat pain, speed of symptom control, and physicians' thoroughness and attention to patient's description of symptoms,^21^, ^22^ compared to the CARES‐MIS, which contains only one item specifically on symptom control.^23^ Those with a higher symptom burden may improve most for these symptom‐related items.

Interpretation of our study findings may be considered in the context of the minimal clinically important difference (MCID) for health‐related quality of life measures. Although no specific MCID has been established for any of the measures in the present study, some may be extrapolated from other quality of life measures. In that regard, a 5–10% change in quality‐of‐life scores is generally considered to be significant.^30^, ^31^, ^32^, ^33^, ^34^ A change in FACIT‐Sp total score of 8.25 points (5.2%) from baseline was used in the original cluster‐randomized controlled trial to calculate the sample size, based on evidence supporting this as the MCID.^30^, ^31^, ^32^, ^33^, ^34^ In our study, the change in FACIT‐Sp scores observed among those with high baseline symptoms was found to be above the MCID, with a mean change of 8.7 points from baseline, supporting the clinical significance of our findings. For our secondary quality‐of‐life outcome, the mean change in QUAL‐E score was 4.2 points (4%) from baseline. While this is less than the 5% change estimated as an MCID for quality‐of‐life measures, a validated MCID for QUAL‐E has yet to be determined and MCID values may be subject to change based on study population and context.^35^ In general, these results are consistent with those of reviews and meta‐analyses demonstrating that effect sizes for the impact of palliative care on quality of life are significant but small.^4^, ^6^, ^9^ No MCID values are available for satisfaction with care or clinician‐patient interactions.

Various consensus‐based criteria have been proposed to prioritize referrals by primary and secondary providers to specialized palliative care. These criteria tend to fall into the general categories of physical symptoms, cancer trajectory, prognosis, performance status, psychosocial distress, and end‐of‐life care planning.^13^, ^36^ However, there is a need for evidence to establish clearly defined criteria to identify the patients most likely to benefit from early palliative care intervention. Such criteria are most likely to be utilized if they are based on symptoms or functions that are already routinely assessed or can be assessed relatively easily in clinical practice. Symptom screening is already routinely conducted in many oncology settings^10^, ^12^, ^37^ and could be linked to triaged early palliative care rather than relying on oncologist referral.

This study has limitations. While it stems from a sound clinical rationale, the subgroup analysis was unplanned, and the study was not powered for this analysis. Generalizability is limited as participants were mainly of European ethnicity, English‐speaking, well‐educated, and receiving treatment in a large, urban, comprehensive cancer center. The confidence intervals reported in this study are relatively wide; this is likely due to splitting the sample into two separately analyzed groups, which reduced power. Selection bias due to randomization of clusters before consent of individuals is a limitation of cluster‐randomized trials, including this study.^3^, ^38^, ^39^ Blinding of those in the intervention group was impossible, although neither trial arm was aware of the existence of the other.^3^ As well, the attrition rate in the current study was higher in the high baseline symptom group compared to the low baseline symptom group. Although this is expected as those with a higher symptom burden might be more likely to drop out or die, it may affect the validity of our findings. Nevertheless, our sensitivity analyses performed with this limitation in mind were broadly consistent with the main findings.

Overall, our study findings suggest that symptom severity may be an appropriate screening mechanism to identify patients who would most benefit from early palliative care and to trigger early palliative care referrals. Further trials with longer follow‐up are needed to assess the feasibility and effectiveness of a triaged model of early palliative care.

## CONFLICT OF INTEREST

We declare that we have no competing conflicts of interest.

## AUTHOR CONTRIBUTIONS

Rebecca Rodin: Conceptualization and design, analysis and interpretation of data, writing and critical revision of the report. Nadia Swami: Acquisition and interpretation of data, critical revision of the report, administrative support. Ashley Pope: Interpretation of data, critical revision of the report, administrative support. David Hui: Interpretation of data and critical revision of the report. Breffni Hannon: Interpretation of data and critical revision of the report. Lisa Le: Conceptualization and design, formal analysis and interpretation of data, writing and critical revision of the report. Camilla Zimmermann: Conceptualization and design, analysis and interpretation of data, writing and critical revision of the report. All authors have provided final approval of the version to be published and have agreed to be accountable for all aspects of the work.

## ETHICS STATEMENT

This study was performed in line with the principles of the Declaration of Helsinki. Approval was granted by the University Health Network Research Ethics Board (#06‐0525‐CE).

## Funding information

This study was funded by the Canadian Cancer Society (CZ; grant number 700862) and the Canadian Institutes of Health Research (CZ; grant number 152996).

## CLINICAL TRIAL REGISTRATION

ClinicalTrials.gov Identifier: NCT01248624.

## Data Availability

The data that support the findings of this study are available from the corresponding author upon reasonable request.
